# Prediction of the Cochlear Implant Electrode Insertion Depth: Clinical Applicability of two Analytical Cochlear Models

**DOI:** 10.1038/s41598-020-58648-6

**Published:** 2020-02-24

**Authors:** G. Mertens, V. Van Rompaey, P. Van de Heyning, E. Gorris, V. Topsakal

**Affiliations:** 1 Univ. Department Otorhinolaryngology, Head & Neck Surgery, Antwerp University Hospital, Antwerp, Belgium; 2 Faculty of Medicine and Health Sciences, Antwerp University, Antwerp, Belgium; 3 Department information and communications technology (ICT), Antwerp University Hospital, Antwerp, Belgium

**Keywords:** Health care, Bone imaging, Tomography

## Abstract

Although the spiral anatomy of the human cochlea seems evident, measuring the highly inter-variable true dimensions is still challenging. Today, only a few three-dimensional reconstruction models of the inner ear are available. Previously, spiral equations were applied to two-dimensional computed tomography (CT) images to predict the electrode insertion depth prior to cochlear implantation. The study aimed primarily to compare the clinical applicability of two analytical cochlear models using a recently introduced planning software to predict the insertion depth of the electrode array of 46 cochlear implant recipients. One was based upon the Escudé formula, which relies only on the basal turn diameter, and another based upon the Elliptic-Circular Approximation (ECA), using the diameter and width. Each case was measured twice by two ENT surgeons. Secondly, in order to measure the benefit of the new planning software over the use of the existing clinical routine method, the results were compared to the prediction based upon a two-dimensional CT image. The intra -and inter-observer agreement using the planning software was significantly better when the ECA was applied, compared to the Escudé formula (p < 0.01). As a reference, the predicted insertion depth was compared to the actual insertion depth measured on post-operative images. The mean absolute error was |2.36| (|1.11|) mm in case of the Escudé approach and |1.19| (|0.92|) mm in case of the ECA. The use of a new planning software that allows three-dimensional handling, integrating the diameter and width of the basal turn (ECA formula), resulted in the most accurate predictions of the electrode insertion depths.

## Introduction

Cochlear implantation is the current standard for the treatment of severe-to-profound sensorineural hearing loss. Despite proliferation of cochlear implant (CI) technology, implantation remains a surgical procedure that requires specific preparation. In order to prepare the surgical procedure, a large number of surgeons create a mental map of the patient’s anatomy based upon interpreting two-dimensional computed tomography (CT) slices acquired preoperatively. The surgeon interprets the CT slices and tries to estimate the cochlear duct length (CDL), i.e. the length to accommodate the electrode array. This is not a trivial task, since the cochlea is highly variable in size^1,2^. It is hypothesized that preoperative estimation of the CDL can be used to provide surgeons with valuable information to assist with the selection of suitable electrode arrays. As we move towards atraumatic full electrode insertions in an attempt to preserve hearing and structures, the selection of the electrode array is crucial. By selecting an electrode array that optimally fits the cochlear anatomy of the patient, over-insertions can be avoided allowing preservation of cochlear structures^3,4^. Today, maximum structure preservation has become an important goal during cochlear implantation, since increasing research has proven that cochlear trauma limits the effectivity of electrical stimulation^5,6^. Avci *et al*. successfully provided a high-resolution visualization of the internal dimensions and the variability of different morphologies of the cochlea with a precision exceeding that of previous investigations. The described dimensions can be used to investigate the ideal location of the electrode array within the scala, with particular emphasis on the insertion depth of the implant^7^. Moreover, based upon previous literature, better speech understanding is expected in cases with deeper electrode insertions and should therefore be pursued^8,9^. The results of Buchman *et al*. suggest that deeper electrode insertions (and greater insertion angles) appear to offer better speech perception in the early post-activation period. They speculate that deeper electrode insertion angles produce greater degrees of cochlear coverage in the apical regions and therefore better tonotopic place representation for stimulation^9^.

As presented in the review of Koch *et al*., there have been a large number of studies introduced from 1884 to the present day that explicitly measured the CDL^10^. These methods have been measuring at both the bony lateral wall (LW) and at the level of the Organ of Corti (OC). The first method that was used to evaluate the CDL, was the *direct* method. This method measures the length from histologic sections by a micrometer under a microscope^2,11^. Later, consistent protocols were developed to graphically represent the CDL by using landmarks from histological sections or plastic casts^12–14^. However, this *indirect* method has some major drawbacks. An important drawback of using two-dimensional slices is the cutting angle effect. This effect is known as the effect of not choosing the correct plane for two-dimensional graphical reconstruction, which causes a false representation of the cochlear dimensions. Moreover, indirect measurements were found to underestimate the CDL, since they do not include the unique shape of the hook region^15^. Three-dimensional reconstructions on the other hand, consider the entire three-dimensional shape of the cochlea. Since they are not susceptible to the viewing angle effects, they are the most accurate models to measure the CDL^10,16–18^. Up until today, the reconstruction of such three-dimensional models has to be performed by trained experts and is too costly and complex to be integrated in clinical routine. The quality of the current cochlear clinical images is also not sufficient for a consistent three-dimensional reconstruction of the cochlea. To acquire better resolution, extra scanning or specific protocols are required^19^, which are not only time-consuming but also involve extra radiation exposure.

The basal turn of the cochlea is known as the most visible part on clinical CT images^20^. Therefore, spiral equations have been developed to rely on measurements of the basal turn parameters. Escudé *et al*. defined a logarithmic equation to determine the CDL at the level of the lateral wall at each cochlear angle θ using a single linear measurement of basal turn diameter A^21^. Alexiades then adopted the Escudé formula to estimate the CDL at the level of the electrode array (CDL_i_)^22^. Since the Escudé formula assumes a linear dependency of A and B, it does not account for the basal turn width B. However, a study of Meng *et al*. revealed that the ratio of A and B is not consistent but may vary substantially^23^. Therefore, Schurzig *et al*. introduced a new Elliptic-Circular Approximation, which uses both diameter A and width B of the cochlear basal turn^20^. *Another key cochlear parameter that can affect the prediction outcomes is the height of the scala. However, due to the poor resolution of the clinical CT this measurement is not possible*. To be able to investigate these analytical cochlear models, there is a need for a planning software that allows three-dimensional handling to provide a quick individualized oblique view of the cochlea based on clinically available CT images. Therefore, it should be easy to make decisions in advance and to plan the best possible outcome for each CI candidate. Recently, a new commercial otological planning software with special focus on cochlear implantation has been introduced^24^.

The primary aim of the retrospective single-centre study was to investigate the clinical applicability of two analytical cochlear models using a recently introduced planning software that allows three-dimensional handling to predict the electrode insertion depth prior to cochlear implantation. Secondly, the results were compared to the prediction using the traditional estimation based upon the A measurement on two-dimensional graphical CT reconstructions. Moreover, the predicted electrode insertion depths were validated by comparing them to the post-operative actual insertion depth.

## Study Design

An experimental version of the planning software was used to analyse the existing pre- and postoperative CT images of 49 CI recipients of the Ear, Nose, and Throat (ENT) department of the Antwerp University Hospital, Belgium. The corresponding datasets were analysed by two experienced ENT surgeons, trained specifically in computer-assisted planning. The study was conducted in accordance with the recommendations of the ethics committee of the Antwerp University Hospital and the protocol was approved on September 9^th^ 2017 (protocol number 17/35/395). The committee waived the need to obtain informed consents for this study.

### Subjects

Subjects were selected if they were implanted with the FLEX28 electrode array (MED-EL, Innsbruck, Austria) and if there were pre- and postoperative CT images of their cochleae available. 49 consecutive CI candidates were screened for the study. After a preliminary screening of pre- and postoperative CT images, one otosclerosis case (Subj. 22) was excluded for further analysis because the location of the round window could not be optimally determined due to significant ossification. Subject 29 and subject 49 were also excluded for further analysis since, although complete insertion was predicted preoperatively, post-operative images exposed partial electrode insertion. Of the 46 remaining cases, 21 were male and 25 were female. The median age at the day of the preoperative CT scan was 56 years old ranging from 6 to 81 years. The entire overview of the included subjects (ear of implantation, gender and age at implantation) can be found as Supplementary Table A.

### Statistical analysis

Using data from previous studies, the expected standard deviation of the measured linear insertion depth is ±1.5 mm^10^. Using a non-parametric (Wilcoxon) test, a sample of 42 cochleae would be needed to show that the mean determined by this study is inside this insertion depth range of the previous studies at a power of 90% (significance level of p = 0.05). To investigate the differences between the Escudé approach and ECA nonparametric Wilcoxon signed-rank tests were used. Degree of agreements between both observations (observation 1 and observation 2) and between both observers were analysed using intra-class correlation coefficients (ICC). IBM SPSS Statistics version 24 (IMB; Armonk, NY) was used for the statistical analyses.

## Methods

### Pre-operative intra and inter-observer agreement

The present study used the clinically available DICOM files (0.1875 * 0.1875 * 0.3 mm voxel), which are part of the clinical pre- and postoperative test protocol in CI. The intra- and inter-observer agreement analysis was performed on the pre-op measurements of the cochlear parameters and the electrode insertion depth predictions. The intra-observer agreement is defined as the repeatability or in ability of the same observer to come up with the same result on a second measurement performed on the same sample. Therefore, each observer measured the parameters two times, with at least an interval of one week between the two measurements. The inter-observer agreement on the other hand, is defined as the reproducibility of the measurements. To evaluate the extent of agreement, intraclass correlation coefficients (ICC) were calculated and Bland-Altman plots were created.

### Equations used to predict electrode insertions depths

**Figure 1:**
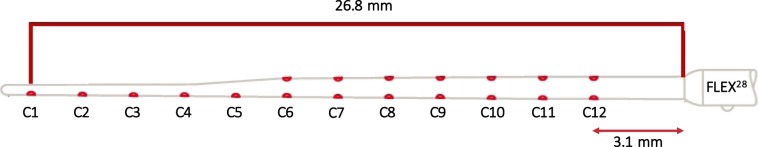
Flexible 28 mm electrode array (MED-EL, Innsbruck, Austria). The presented electrode array has a length of 28 mm from the silicone tip to the stopper. The distance between the first contact (C1, left side) and the stopper (ride side) is 26.8 mm.

Typically, the insertion depth of the electrode refers to the covered angular (deg) and linear (mm) length of the cochlea at the electrode silicon tip. The electrode used in the study has a length of 28 mm from the silicon tip to the stopper (Fig. 1). Since this silicon tip is not visible in the post-op images, the validation of the electrode insertion depth prediction was performed on the 1^st^ contact (C1), which is located 26.8 mm from the stopper (1.2 mm from the silicon tip). Therefore, the linear insertion depth of the electrode is taken as 26.8 mm for the flexible 28 mm lateral wall electrode array.

To predict the angular insertion depth of the electrode at C1 the adopted Escudé and ECA formulas were used. Both formulas project the full length of the electrode array (from stopper to the C1) to the estimated cochlear length and assume the electrode would follow a consistent path parallel to the level of the lateral wall, called CDL_i_.

The first approach used an adopted version of the Escudé formula^21^. The original Escudé formula is based on the cochlear diameter (A) and estimates the length of the cochlear lateral wall at each specific angle (Ɵ). The adopted formula for electrode insertion length prediction implemented in the software assumes that the lateral wall electrode lies under the organ of Corti, 0.5 mm away from the lateral wall and therefore 1 mm is subtracted from the A value to accommodate for this distance (Eq. 1)^22^.

1 $$CD{L}_{i}({\varTheta})={p}_{1}(A-2\ast 0.5)\mathrm{ln}(1+{\varTheta}/{p}_{2})\,with\,{p}_{1}=2.62,\,{p}_{2}=235^\circ $$

**Figure 2:**
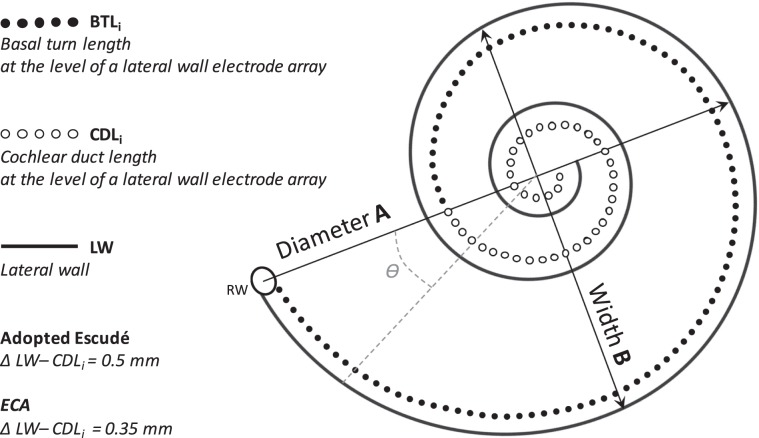
Schematic representation of the parameters of the cochlear duct. The lateral wall (LW), the Basal turn length (BTL) and the Cochlear duct length (CDL) at the level of a LW electrode array are shown. The arrows show the diameter (**A**) and the width (**B**) of the basal turn. The adopted Escudé approach uses 0.5 mm for the distance between the LW and the LW electrode array, while ECA uses 0.35 mm.

The second approach used the recently introduced Elliptic-Circular Approximation (ECA)^20^. In contrast to the Escudé formula, this estimation does not only rely on a single linear measurement of the basal turn diameter (A), but also includes the width of the basal turn (B). ECA assumes that the electrodes lie at a level of 0.35 mm from the lateral wall and therefore subtracts a 0.7 mm from the diameter and width (Fig. 2)^22^. In this formula, the basal turn length of the cochlea at the level of the electrode (BTL_i_) is estimated (Eq. 2), and then a percentage-based approximation is used to estimate the insertion depth of the electrode for each angle (Ɵ) (Eq. 3). Supplementary Table B presents for each insertion angle the corresponding basal turn length percentage (1–900 degrees in 1 degree steps). The derivation of the p_BTL_ values is a result of a polynomial fit of the mean BTL_LW_ and CDL_LW_ values of 20 muCT datasets, which is described in detail in Schurzig *et al*. (Eq. 4)^20^.

2 $$BT{L}_{i}=1.18(A-2\ast 0.35)+2.69(B-2\ast 0.35)-\surd (0.72(A-2\ast 0.35)(B-2\ast 0.35))$$

3 $$CD{L}_{i}({\varTheta})={p}_{BTL}({\varTheta})\ast BT{L}_{i}$$

4 $${p}_{BTL}=8.3\ast {10}^{-8}\ast {{\varTheta}}^{3}-2.4\ast {10}^{-4}\ast {{\varTheta}}^{2}+3.4\ast {10}^{-1}\ast {\varTheta}+3.7$$

### Measurement using planning software

**Figure 3:**
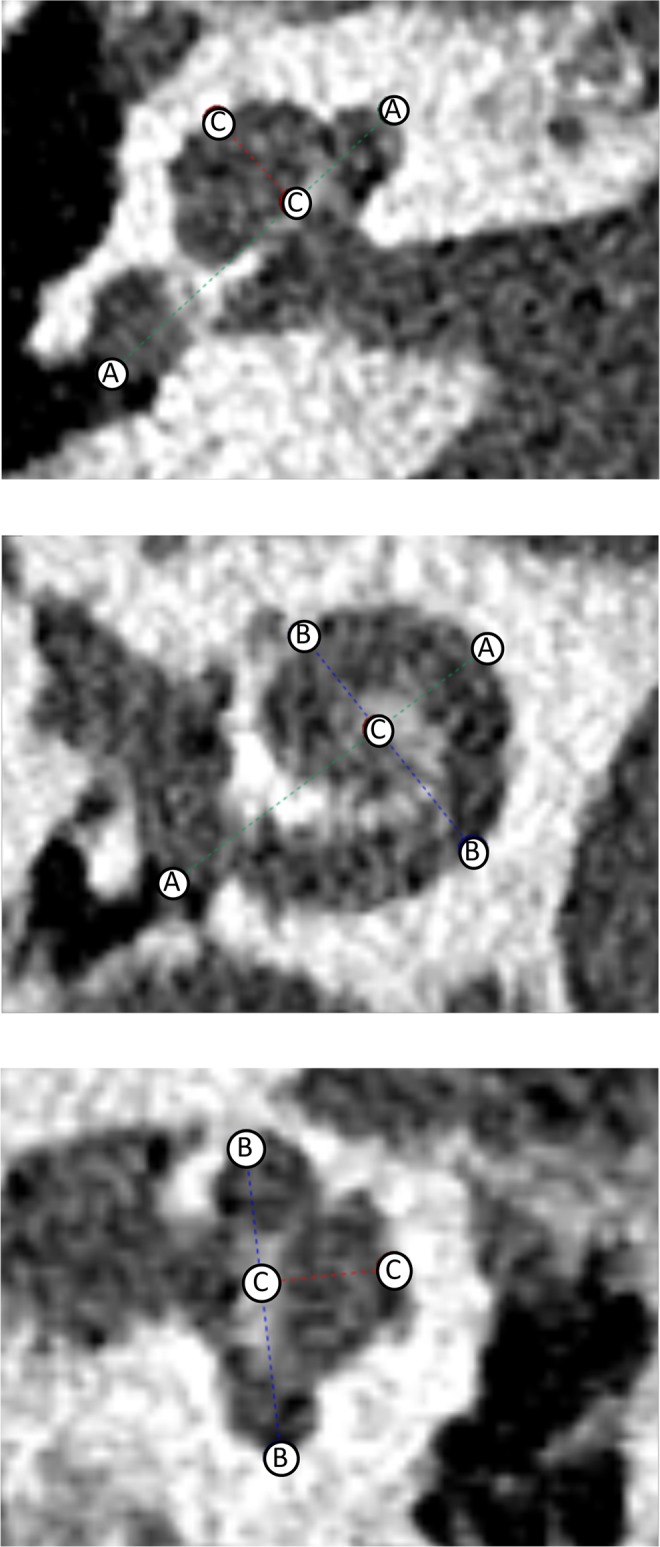
Measurement of the cochlear parameters on the defined optimal cochlear view using the new planning software. Cochlear diameter (**A**), width (**B**) and height (**C**) are shown.

CTs were either imported automatically into the planning software through the PACS network of the hospital or imported from a memory stick. At first, the coronal oblique view, typically referred to as cochlear view, was created by three-dimensionally rotating against the body planes (axial, coronal, and sagittal). This three-dimensional handling is one of the main improvements of using the new planning software compared to the traditional estimations using 2D oblique views. Subsequently, the cochlear parameters of diameter (A) and width (B) were measured by two experienced ENT surgeons on the defined optimal cochlear view. The cochlear diameter (A) is defined as the linear measurement from the round window to the furthest point on the opposite wall of the cochlea, passing through the helicotrema. The cochlear width (B) is defined as the linear measurement perpendicular to the diameter passing through helicotrema connecting the two opposite lateral wall points (Fig. 3).

### Measurement using traditional 2D oblique view

**Figure 4:**
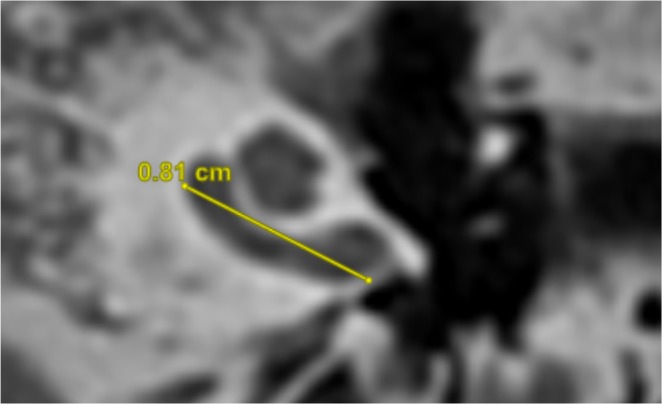
Example of a measurement of the A value on the traditional 2D oblique view in PACS viewer. Cochlear diameter, known as the A value is shown.

To investigate the improvement of using the new planning software, the inter- and intra-observer agreement was compared to the agreement achieved with the previously used traditional A measurement, performed on the oblique slices in the Picture Archiving and Communicating System (PACS) viewer (Fig. 4).

### Postoperative validation of electrode insertion depth prediction

**Figure 5:**
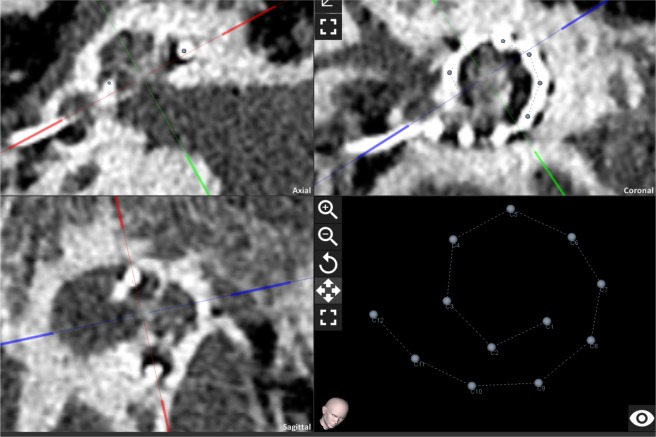
Postoperative tool of the planning software. User defined cochlear view and coordinate system by blue, green and red crosshairs and selected centre point of each electrode contact in the planning software.

The postoperative CT images were used for the evaluation of the actual insertion status of the implanted electrode arrays inside the cochlea. In the first step, the cochlear view and coordinate system was set at the planning software using the cross-hairs and then both observers manually selected a point at the centre of each electrode contact (Fig. 5). The software then uses the user defined coordinate system to estimate the angular insertion depth of the selected centre of each electrode contact. In the next step the actual angular insertion angle of C1 is implemented in Eqs. 1–4 to calculate the actual linear insertion length of C1. To estimate the actual insertion depth of the electrode tip, 1.2 mm is added to the linear insertion depth of C1 (Eq. 5). For the subsequent conversion to the angular insertion depth at the tip, Eqs. 1–4 are used. To assess the accuracy of the predicated angular and linear insertion error the predicted values were subtracted from the actual insertion depth values (Eq. 6). The absolute values are used to assess the mean and standard deviation of the prediction errors for each formula.

5 $$Actual\,linear\,insertio\,depth\,@tip=Actual\,linear\,insertion\,depth\,@C1+1.2\,mm$$

6 $$Prediction\,error\,@C1=Actual\,insertion\,@C1-Predicted\,insertion\,@C1$$

## Results

### Intra and inter-observer agreement

#### Using planning software

**Figure 6:**
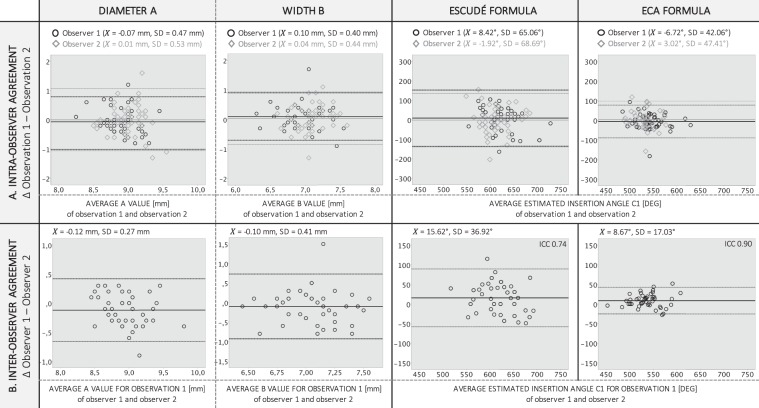
Bland-Altman plots of the intra -and inter-observer agreements for the preoperative A and B measurements and the predictions of the electrode insertion angles, using the adopted Escudé approach and ECA. (**A**) INTRA-OBSERVER AGREEMENT. For each preoperative measurement the differences between observation 1 and observation 2 are shown against the respective averages of both observations. Data from observer 1 are displayed in black and from observer 2 in grey. The solid lines represent the mean differences between both observations. The top dotted lines show the upper 95% limit of agreement, and the bottom dotted lines show the lower 95% limit of agreement (2STDEV). (**B**) INTER-OBSERVER AGREEMENT. For each preoperative measurement the differences between observer 1 and observer 2 for their first observation are shown against the respective averages of both observers. The solid lines represent the mean differences between both observers. The top dotted lines show the upper 95% limit of agreement, and the bottom dotted lines show the lower 95% limit of agreement (2STDEV).

***Intra-observer agreements*** of the preoperative predictions of A, B and the insertion depths of the electrode tip using the planning software are shown in the Bland-Altman in Fig. 6A for each observer. The mean difference of the A measurements at observation one and observation two was −0.07 (SD 0.47) mm for observer one and 0.01 (SD 0.53) mm for observer two. The mean difference of the B measurements at observation one and observation two was 0.10 (SD 0.40) mm for observer one and 0.04 (SD 0.44) mm for observer two. The standard deviation of the differences between the two observations reduced significantly (Wilcoxon Signed Rank test, p < 0.01) when ECA was used (SD_OBSERVER_1_ 42.06°, SD_OBSERVER_2_ 47.41°) for the prediction of the electrode insertion depth at C1 compared to the Escudé approach (SD_OBSERVER_1_ 65.06°, SD_OBSERVER_2_ 68.69°).

***Inter-observer agreements*** of the preoperative predictions of A, B and the insertion depths of the electrode tip using the planning software are shown in the Bland-Altman in Fig. 6B. The mean difference between both observers was −0.12 (SD 0.27) mm for the A measurements and −0.10 (SD 0.41) mm for the B measurements. The standard deviation of the differences between the two observers reduced significantly (Wilcoxon Signed Rank test, p < 0.01) when ECA was used (SD 17.03°) for the prediction of the electrode insertion depth at C1 compared to the Escudé approach (SD 36.92°). The corresponding ICC for the Escudé approach was 0.74, indicating *substantial* agreement rate. The ICC for the ECA was 0.90, indicating an *almost perfect* agreement rate.

#### Using traditional 2D oblique view

The mean difference of the A measurements at observation one and observation two (intra-observer agreement) was 0.09 (SD 0.36) mm for observer one and −0.08 (SD 0.33) mm for observer two. The standard deviation of the prediction of the electrode insertion depth using the traditional 2D oblique view was significantly higher (SD_OBSERVER1_ = 83.97 and SD_OBSERVER2_ = 54.67) compared to the predictions with the planning software.

The mean difference between both observers (inter-observer agreement) was −0.65 (SD 0.48) mm for the A measurements. The standard deviation of the differences between the two observers was 97.32° for the prediction of the electrode insertion depth at C1, resulting in a corresponding ICC of 0.58, indicating *moderate* agreement.

### Validation of electrode insertion depth prediction

The average of the actual angular insertion depth of the tip of the electrode using the planning software for both observers, was 562° (1.56 turns) and 572° (1.58 turns) using the ECA and the Escudé formula respectively. The pre-operative estimation of the angular insertion was the most accurate when the planning software used the ECA formula (573°), compared to the use of the Escudé formula in the software (668°) or the estimation based upon the 2D oblique view (795°). For all three estimation methods the preoperative measures overestimated the angular insertion depth.

In Table 1 the absolute angular and linear insertion depth prediction errors for both observers using the planning software (Escudé and ECA approaches) and the traditional estimation based upon a 2D oblique view are shown. It can be seen from the results that all measures overestimated the insertion depth of the electrode, although, when the B value was added (ECA) using the planning software, the overestimation of the predicted insertion angle reduced compared to the Escudé approach. For observer 1 the mean absolute angular and linear insertion error using the planning software was recorder as |98.99|° (SD |42.70|) and |2.50| mm (SD |1.06|) using the Escudé method and reduced significantly to |39.42|° (SD |30.49|) and |1.30| mm (SD |0.98|) using the ECA method. For observer 2 the absolute angular and linear insertion error using the planning software was recorder as |86.51|° (SD |44.52|) and |2.22| mm (SD |1.16|) using the Escudé method and reduced significantly to |32.55|° (SD |26.63|) and |1.10| mm (SD |0.87|) using the ECA method. Significant higher errors were found when the predictions were performed on 2D oblique views. The entire overview of the raw data of the predicted and the actual angular insertion depth of the electrode tip for observer 1 and 2 can be found as Supplementary Table A. For each observer the results are calculated for both observation 1 and observation 2 using both the adopted Escudé formula and using ECA.

**Table 1:** Absolute angular and linear insertion depth prediction errors for the Escudé approach and ECA for both observers.

Insertion prediction error at contact 1	OBSERVER 1			OBSERVER 2		
	Planning software		2D oblique	Planning software		2D oblique
	Escudé	ECA	Escudé	Escudé	ECA	Escudé
Angular [deg]	|98.99|SD |42.70|	|39.42|SD |30.49|	|275.26|SD 88.61	|86.51|SD|44.52|	|32.55|SD |26.63|	|135.39|SD 63.86
Linear (mm)	|2.50|SD |1.06|	|1.30|SD |0.98|	|5.46|SD 1.35	|2.22|SD |1.16|	|1.10|SD |0.87|	|3.21|SD |1.34|

Accuracy of the predicted angular (DEG) and linear (mm) insertion depth errors for both the planning software (using Escudé approach and ECA) and for the traditional estimation based upon a 2D oblique view. Preoperative predicted values were subtracted from the postoperative actual insertion depth values. Mean errors and their standard deviation (SD) of the absolute values are presented.

## Discussion

The planning software used in the present study was found to be feasible to pursue optimal cochlear views on preoperative clinical CT images. The Elliptic-Circular approximation, which uses both diameter A and width B of the cochlear basal turn, resulted in more accurate predictions of the electrode insertion depth, compared to Escudé approach that relies on the single linear A measurement. This is in agreement with the findings of MENG *et al*. that confute the linear dependency of the cochlear basal turn diameter and width^23^. Moreover, the study of Pietsch *et al*. demonstrated that the geometry is not similar to a logarithmic spiral and that there are genuine inter-individual differences in cochlear geometry. They argue that logarithmic models are good enough to predict the metric length of the cochlear duct, but fail to predict the details of the spiral shape. Therefore, they developed a polynomial model that requires assessment of the basic individual parameters of cochlear base width, length and their intersection by the modiolus^25^.

Previous reconstruction models were too complex and therefore not suitable to complete within the available clinical timeframes. In 2017, van der Jagt *et al*. investigated in a large cohort an automatic tracing method based on voxel intensity to measure cochlear morphology *in vivo* up to and including the second turn of the cochlea. The possible benefit of the automatic method is the accuracy of the method and susceptibility for observer variability because of the use of 180 measurements per cochlear turn instead of four and because of its objectivity^26^. Such an automatic tracing method has a lot of potential in the near future to evaluate CI candidates preoperatively. Another methodology for measuring the cochlear duct length was introduced and evaluated by Würfel *et al*.^27^. This method uses clinically available high-resolution imaging modalities and relies on manually setting a three-dimensional spiral along the outer cochlear wall. However, up until today such methods are not clinical available yet. With the present planning software on the contrary, the preoperative planning takes on average five minutes and the postoperative analysis takes on average 15 minutes, as it takes longer to select all the electrode contacts on the image. So far, pre- and postoperative planning is only possible in normal anatomies. More research is needed to investigate the applicability and validity of the equations in malformed cochleas. The application may prove to be useful for electrode selection in dysplasia cases.

The intra-user variability largely depends on the image resolution. The consistency of the manual measurements performed in the study are expected to improve with increasing image resolution or when the planning software would be able to automatically provide the cochlear parameters. However, the identification of structures such as the round window membrane requires interpretations that can hardly be automated.

As shown in Table 1, all approaches resulted mainly in overestimations of the predicted insertion depths compared to the reference postoperative measurements, with only a small overestimation for the ECA method (OBSERVER1 = 39° and OBSERVER2 = 33°). A similar study performed by Rathgeb *et al*. used the adopted Escudé approach in the planning software. They reported a higher overestimation of 73 (96) degrees^28^. There are certain assumptions made during the pre-op electrode insertion depth prediction that can largely affect the prediction outcomes. Both formulas project the full length of the electrode over a consistent path along the lateral wall, 0.35 mm is case of ECA and 0.50 mm in case of Escudé. This presupposes that the application is for mid-range and lateral wall electrodes and not for modular hugging electrodes. One could argue that the higher overestimation rate found for the Escudé approach is due to the fact that (2*0.5) mm is subtracted from the A value^22^, whereas for ECA this was only (2*0.35) mm. However, only a small reduction of the overestimation was observed when using (2*0.35) instead of (2*0.5) in the Escudé approach as well. The angular insertion prediction error improved from 99.80° (SD 49.58) to 63.03° (SD 37.71) for observer one and from 84.19° (SD 47.86) to 55.13° (SD 34.39) for observer two. Both formulas also assume a full insertion of the electrode at which the stopper of the electrode array is exactly located at the level of the round window. They do not accommodate for the diameter of the scala, micro blockages inside the cochlea, surgeon’s preference or any other clinical reasons that would cause the electrode to bend. Another assumption made in the analytical cochlear models is the fixed position of the stopper at the level of the round window. However, the conical stopper is inserted up to the level of optimal ceiling. Since this is highly dependent of the anatomy of the round window niche, the distance between the stopper and the round window can cause a prediction error up to 1–2 mm. The ECA underestimates (one-tailed) the insertion depth of a lateral wall electrode on average with −0.58 mm. Taking into account that mean prediction error is about 1.19 mm, only 16% of the planned insertions will exceed the predicted insertion depth and only 2.5% will exceed it with more than 2.38 mm. Therefore, ECA offers a reasonable reliable tool to determine the electrode insertion depth, and it is up to the surgeon to decide on which limits are important i.e. whether not to pass a predefined depth, as is the case in residual hearing or to reach a least a certain insertion depth as in the case of severe hearing loss over all frequencies.

In addition to preoperative insertion predictions, the postoperative tool of the software allows the identification of the exact electrode location within the cochlea which would enable tonotopic stimulation^15^. This could optimize fitting strategies for CI patients and allow a preoperative or even pre-set fitting strategy based upon a postoperative CT scan to shorten the rehabilitation time with audiologists and speech therapists. Recent studies on CIs have suggested that speech perception is optimized when the frequency information is presented to the normal acoustic tonotopic cochlear location^17,18,29,30^. Frequency-place maps that are shifted or distorted relative to the normal tonotopic map may reduce speech recognition.

In conclusion, predictions of the electrode insertion depth were made using an experimental version of a new otological planning software, which allows guided three-dimensional handling. The ECA that is based upon two basal turn parameters (diameter and width) yielded more accurate predictions of the electrode insertion depth, compared to the adopted Escudé approach which is based upon a single diameter measurement.

## Supplementary information

Supplementary information

Supplementary information2

## Data Availability

The datasets generated in this study are available from the corresponding author on reasonable request.
