# Supplementary material for: Prediction of the Cochlear Implant Electrode Insertion Depth: Clinical Applicability of two Analytical Cochlear Models
Source: Sci Rep. 2020 Feb 24;10:3340. doi: 10.1038/s41598-020-58648-6 (PMC7039896; doi:10.1038/s41598-020-58648-6)
Supplement: Supplementary file 1 — Supplementary information [file 41598_2020_58648_MOESM1_ESM.docx]

# Prediction of the cochlear implant electrode insertion depth: clinical applicability of two analytical cochlear models

*G. Mertens^1,2,*^, V. Van Rompaey^1,2^, P. Van de Heyning^1,2^, E. Gorris^3^, and V. Topsakal^1,2^*

^1^ Univ. Dept. Otorhinolaryngology, Head & Neck Surgery, Antwerp University Hospital, Belgium.

^2^ Faculty of Medicine and Health Sciences, Antwerp University, Belgium.

^3^ Dept. information and communications technology (ICT), Antwerp University Hospital, Belgium.

*** Address correspondence to**

**griet.mertens@uza.be**

Antwerp University Hospital

Univ. Dept. Otorhinolaryngology, Head and Neck Surgery

Griet Mertens

Wilrijkstraat 10

2650 Edegem, Belgium

0032 38 21 32 45

### **Additional Information**

*Competing information:*

*Financial disclosure: The Antwerp University Hospital is currently receiving a research grant from MED-EL (Innsbruck, Austria). Non-Financial disclosure: None.*

**Table A online. Overview subjects, including raw data of the predicted and actual insertion angles of the electrode tip.**

*For each included case implanted ear (R, Right and L, Left), gender (F, Female and M, Male) and age at implantation (years) are presented. Raw data of the predicted and the actual angular insertion depth of the electrode tip is given for observer 1 and 2. For each observer, results are calculated for both observation 1 (O1) and observation 2 (O2) using the adopted Escudé formula and using ECA.*

| NR | Ear | Gender | Age at implantation |  |  | **OBSERVER 1** | | | | | | | |  |  | **OBSERVER 2** | | | | | | | |
| --- | --- | --- | --- | --- | --- | --- | --- | --- | --- | --- | --- | --- | --- | --- | --- | --- | --- | --- | --- | --- | --- | --- | --- |
|  |  |  |  |  |  | Preoperative Estimation Insertion Angle (°) | | | | Actual Postoperative  Insertion Angle (°) | | | |  |  | Preoperative Estimation Insertion Angle (°) | | | | Actual Postoperative  Insertion Angle (°) | | | |
|  |  |  |  |  |  | Escudé | | ECA | | Escudé | | ECA | |  |  | Escudé | | ECA | | Escudé | | ECA | |
|  |  |  |  |  |  | O1 | O2 | O1 | O2 | O1 | O2 | O1 | O2 |  |  | O1 | O2 | O1 | O2 | O1 | O2 | O1 | O2 |
| 001 | R | M | 36 |  |  | 658.5 | 658.5 | 564.0 | 573.0 | 535.2 | 535.2 | 527.0 | 527.0 |  |  | 643.9 | 658.5 | 561.0 | 514.0 | 534.6 | 535.2 | 527.0 | 525.0 |
| 002 | R | F | 59 |  |  | 658.5 | 603.5 | 573.0 | 543.0 | 548.4 | 546.3 | 541.0 | 539.0 |  |  | 689.7 | 673.8 | 562.0 | 550.0 | 549.5 | 549.0 | 540.0 | 540.0 |
| 003 | R | M | 76 |  |  | 706.3 | 643.9 | 574.0 | 588.0 | 521.5 | 519.3 | 513.0 | 514.0 |  |  | 643.9 | 741.8 | 552.0 | 589.0 | 519.3 | 522.7 | 513.0 | 514.0 |
| 004 | L | M | 67 |  |  | 706.3 | 591.0 | 583.0 | 486.0 | 592.6 | 588.0 | 581.0 | 576.0 |  |  | 658.5 | 525.3 | 600.0 | 477.0 | 590.7 | 585.0 | 581.0 | 576.0 |
| 005 | R | F | 60 |  |  | 658.5 | 616.4 | 591.0 | 563.0 | 693.4 | 691.5 | 679.0 | 678.0 |  |  | 706.3 | 629.9 | 620.0 | 525.0 | 695.5 | 692.1 | 681.0 | 676.0 |
| 006 | L | F | 60 |  |  | 643.9 | 673.8 | 561.0 | 594.0 | 506.6 | 507.7 | 501.0 | 502.0 |  |  | 603.5 | 689.7 | 560.0 | 553.0 | 505.1 | 508.2 | 501.0 | 501.0 |
| 007 | L | M | 72 |  |  | 723.6 | 706.3 | 634.0 | 592.0 | 594.3 | 593.6 | 584.0 | 582.0 |  |  | 706.3 | 535.3 | 620.0 | 533.0 | 593.6 | 586.6 | 583.0 | 579.0 |
| 008 | R | F | 36 |  |  | 673.8 | 673.8 | 576.0 | 567.0 | 579.7 | 579.7 | 569.0 | 569.0 |  |  | 629.9 | 689.7 | 541.0 | 607.0 | 578.0 | 580.3 | 568.0 | 571.0 |
| 009 | R | F | 68 |  |  | 673.8 | 673.8 | 594.0 | 654.0 | 499.2 | 499.2 | 494.0 | 497.0 |  |  | 673.8 | 706.3 | 567.0 | 611.0 | 499.2 | 500.3 | 493.0 | 495.0 |
| 010 | R | F | 78 |  |  | 706.3 | 616.4 | 574.0 | 581.0 | 557.6 | 554.2 | 548.0 | 548.0 |  |  | 643.9 | 629.9 | 561.0 | 575.0 | 555.2 | 554.7 | 547.0 | 548.0 |
| 011 | R | M | 51 |  |  | 658.5 | 780.6 | 610.0 | 646.0 | 585.4 | 590.0 | 577.0 | 578.0 |  |  | 658.5 | 723.6 | 564.0 | 634.0 | 585.4 | 587.9 | 575.0 | 578.0 |
| 012 | L | F | 80 |  |  | 706.3 | 616.4 | 574.0 | 555.0 | 570.3 | 566.9 | 559.0 | 559.0 |  |  | 673.8 | 658.5 | 576.0 | 556.0 | 569.1 | 568.5 | 560.0 | 559.0 |
| 013 | R | M | 51 |  |  | 579.0 | 616.4 | 513.0 | 546.0 | 523.1 | 524.6 | 517.0 | 518.0 |  |  | 616.4 | 658.5 | 522.0 | 547.0 | 524.6 | 526.2 | 517.0 | 518.0 |
| 014 | R | M | 81 |  |  | 723.6 | 658.5 | 577.0 | 591.0 | 591.1 | 588.6 | 578.0 | 579.0 |  |  | 689.7 | 629.9 | 562.0 | 567.0 | 589.8 | 587.5 | 578.0 | 578.0 |
| 015 | R | M | 65 |  |  | 616.4 | 706.3 | 546.0 | 592.0 | 582.7 | 586.2 | 573.0 | 575.0 |  |  | 603.5 | 689.7 | 560.0 | 571.0 | 582.2 | 585.6 | 574.0 | 574.0 |
| 016 | L | F | 41 |  |  | 723.6 | 673.8 | 595.0 | 559.0 | 606.0 | 604.1 | 593.0 | 591.0 |  |  | 760.7 | 658.5 | 621.0 | 522.0 | 607.3 | 603.4 | 594.0 | 590.0 |
| 017 | R | F | 42 |  |  | 689.7 | 643.9 | 598.0 | 536.0 | 599.4 | 597.6 | 588.0 | 585.0 |  |  | 741.8 | 643.9 | 608.0 | 536.0 | 601.3 | 597.6 | 588.0 | 585.0 |
| 018 | L | F | 70 |  |  | 723.6 | 616.4 | 577.0 | 563.0 | 500.9 | 497.1 | 494.0 | 493.0 |  |  | 673.8 | 643.9 | 576.0 | 588.0 | 499.2 | 498.1 | 493.0 | 494.0 |
| 019 | L | F | 75 |  |  | 673.8 | 658.5 | 594.0 | 582.0 | 585.5 | 584.9 | 575.0 | 575.0 |  |  | 673.8 | 673.8 | 576.0 | 559.0 | 585.5 | 585.5 | 574.0 | 574.0 |
| 020 | L | M | 71 |  |  | 706.3 | 689.7 | 548.0 | 545.0 | 540.1 | 539.5 | 529.0 | 529.0 |  |  | 579.0 | 706.3 | 521.0 | 531.0 | 535.3 | 540.1 | 528.0 | 529.0 |
| 021 | L | F | 73 |  |  | 723.6 | 658.5 | 577.0 | 610.0 | 600.7 | 598.1 | 587.0 | 589.0 |  |  | 741.8 | 723.6 | 580.0 | 568.0 | 601.3 | 600.7 | 587.0 | 587.0 |
| 023 | R | M | 59 |  |  | 643.9 | 706.3 | 561.0 | 583.0 | 522.5 | 524.7 | 516.0 | 517.0 |  |  | 673.8 | 689.7 | 559.0 | 580.0 | 523.5 | 524.1 | 516.0 | 517.0 |
| 024 | L | M | 60 |  |  | 706.3 | 591.0 | 583.0 | 524.0 | 602.1 | 597.5 | 589.0 | 587.0 |  |  | 616.4 | 629.9 | 563.0 | 525.0 | 598.5 | 599.1 | 589.0 | 587.0 |
| 025 | L | M | 64 |  |  | 643.9 | 760.7 | 579.0 | 612.0 | 552.1 | 556.3 | 545.0 | 546.0 |  |  | 616.4 | 760.7 | 563.0 | 612.0 | 551.0 | 556.3 | 544.0 | 546.0 |
| 026 | L | F | 66 |  |  | 689.7 | 658.5 | 589.0 | 610.0 | 628.0 | 626.7 | 614.0 | 615.0 |  |  | 616.4 | 643.9 | 581.0 | 588.0 | 624.9 | 626.1 | 614.0 | 614.0 |
| 027 | R | F | 74 |  |  | 603.5 | 706.3 | 535.0 | 574.0 | 555.8 | 559.7 | 548.0 | 550.0 |  |  | 603.5 | 643.9 | 535.0 | 536.0 | 555.8 | 557.4 | 548.0 | 548.0 |
| 028 | R | M | 66 |  |  | 603.5 | 706.3 | 552.0 | 601.0 | 595.9 | 600.0 | 586.0 | 588.0 |  |  | 629.9 | 643.9 | 558.0 | 536.0 | 597.0 | 597.6 | 586.0 | 585.0 |
| 030 | R | F | 65 |  |  | 741.8 | 706.3 | 608.0 | 641.0 | 671.5 | 670.0 | 655.0 | 656.0 |  |  | 760.7 | 706.3 | 621.0 | 601.0 | 672.2 | 670.0 | 655.0 | 655.0 |
| 031 | L | F | 38 |  |  | 579.0 | 760.7 | 483.0 | 675.0 | 520.0 | 526.5 | 513.0 | 521.0 |  |  | 535.3 | 760.7 | 487.0 | 653.0 | 518.2 | 526.5 | 513.0 | 520.0 |
| 032 | R | M | 17 |  |  | 643.9 | 658.5 | 561.0 | 582.0 | 545.7 | 546.3 | 538.0 | 539.0 |  |  | 658.5 | 673.8 | 547.0 | 585.0 | 546.3 | 546.8 | 537.0 | 539.0 |
| 033 | R | F | 25 |  |  | 689.7 | 658.5 | 598.0 | 582.0 | 570.8 | 569.6 | 561.0 | 561.0 |  |  | 643.9 | 673.8 | 579.0 | 559.0 | 569.0 | 570.2 | 561.0 | 560.0 |
| 034 | R | F | 73 |  |  | 658.5 | 658.5 | 591.0 | 591.0 | 578.0 | 578.0 | 569.0 | 569.0 |  |  | 603.5 | 673.8 | 577.0 | 567.0 | 575.8 | 578.6 | 568.0 | 568.0 |
| 035 | R | F | 52 |  |  | 706.3 | 673.8 | 574.0 | 585.0 | 619.1 | 617.8 | 605.0 | 605.0 |  |  | 673.8 | 643.9 | 567.0 | 588.0 | 617.8 | 616.6 | 604.0 | 605.0 |
| 036 | R | M | 49 |  |  | 673.8 | 567.5 | 542.0 | 495.0 | 589.7 | 585.4 | 577.0 | 575.0 |  |  | 616.4 | 603.5 | 530.0 | 519.0 | 587.4 | 586.9 | 576.0 | 576.0 |
| 037 | R | F | 52 |  |  | 658.5 | 741.8 | 600.0 | 608.0 | 540.5 | 543.4 | 534.0 | 534.0 |  |  | 643.9 | 689.7 | 570.0 | 598.0 | 539.9 | 541.6 | 532.0 | 534.0 |
| 038 | L | M | 61 |  |  | 706.3 | 629.9 | 531.0 | 549.0 | 556.5 | 553.6 | 545.0 | 545.0 |  |  | 706.3 | 616.4 | 523.0 | 522.0 | 556.5 | 553.1 | 544.0 | 544.0 |
| 039 | R | M | 54 |  |  | 643.9 | 616.4 | 597.0 | 563.0 | 657.9 | 656.6 | 645.0 | 644.0 |  |  | 658.5 | 643.9 | 582.0 | 528.0 | 658.5 | 657.9 | 644.0 | 642.0 |
| 040 | L | F | 35 |  |  | 706.3 | 629.9 | 556.0 | 602.0 | 558.6 | 555.8 | 548.0 | 550.0 |  |  | 643.9 | 616.4 | 544.0 | 581.0 | 556.3 | 555.2 | 547.0 | 549.0 |
| 041 | R | F | 67 |  |  | 723.6 | 689.7 | 624.0 | 598.0 | 583.7 | 582.4 | 573.0 | 572.0 |  |  | 689.7 | 567.5 | 627.0 | 576.0 | 582.4 | 577.5 | 574.0 | 571.0 |
| 042 | L | F | 16 |  |  | 706.3 | 706.3 | 574.0 | 611.0 | 531.6 | 531.6 | 523.0 | 524.0 |  |  | 689.7 | 603.5 | 545.0 | 552.0 | 531.0 | 527.8 | 521.0 | 522.0 |
| 043 | R | F | 69 |  |  | 673.8 | 591.0 | 604.0 | 574.0 | 531.5 | 528.4 | 525.0 | 524.0 |  |  | 673.8 | 658.5 | 567.0 | 573.0 | 531.5 | 530.9 | 523.0 | 523.0 |
| 044 | L | M | 76 |  |  | 629.9 | 673.8 | 533.0 | 567.0 | 528.3 | 529.9 | 521.0 | 522.0 |  |  | 591.0 | 689.7 | 532.0 | 536.0 | 526.8 | 530.5 | 521.0 | 521.0 |
| 045 | R | M | 48 |  |  | 658.5 | 591.0 | 539.0 | 557.0 | 562.7 | 560.0 | 552.0 | 553.0 |  |  | 658.5 | 643.9 | 539.0 | 561.0 | 562.7 | 562.1 | 552.0 | 553.0 |
| 046 | L | F | 64 |  |  | 780.6 | 801.5 | 657.0 | 684.0 | 584.6 | 585.4 | 574.0 | 575.0 |  |  | 706.3 | 689.7 | 601.0 | 627.0 | 582.0 | 581.4 | 571.0 | 573.0 |
| 047 | R | M | 65 |  |  | 706.3 | 823.4 | 662.0 | 654.0 | 562.9 | 566.9 | 556.0 | 556.0 |  |  | 760.7 | 723.6 | 631.0 | 586.0 | 564.8 | 563.5 | 555.0 | 553.0 |
| 048 | R | M | 6 |  |  | 689.7 | 689.7 | 598.0 | 598.0 | 659.8 | 659.8 | 645.0 | 645.0 |  |  | 723.6 | 723.6 | 605.0 | 605.0 | 661.2 | 661.2 | 645.0 | 645.0 |
| mean | | | |  |  | **678.3** | **669.0** | **577.5** | **584.5** | **572.2** | **571.8** | **562.3** | **562.5** |  |  | **661.0** | **663.1** | **568.3** | **565.4** | **571.6** | **571.6** | **561.8** | **561.8** |
|  |  |  |  |  |  | **673.6** | | **581.0** | | **572.0** | | **562.4** | |  |  | **662.1** | | **566.8** | | **571.6** | | **561.8** | |
