# Supplementary material for: Prediction of the Cochlear Implant Electrode Insertion Depth: Clinical Applicability of two Analytical Cochlear Models
Source: Sci Rep. 2020 Feb 24;10:3340. doi: 10.1038/s41598-020-58648-6 (PMC7039896; doi:10.1038/s41598-020-58648-6)
Supplement: Supplementary file 2 — Supplementary information2 [file 41598_2020_58648_MOESM2_ESM.docx]

# Prediction of the cochlear implant electrode insertion depth: clinical applicability of two analytical cochlear models

*G. Mertens^1,2,*^, V. Van Rompaey^1,2^, P. Van de Heyning^1,2^, E. Gorris^3^, and V. Topsakal^1,2^*

^1^ Univ. Dept. Otorhinolaryngology, Head & Neck Surgery, Antwerp University Hospital, Belgium.

^2^ Faculty of Medicine and Health Sciences, Antwerp University, Belgium.

^3^ Dept. information and communications technology (ICT), Antwerp University Hospital, Belgium.

*** Address correspondence to**

**griet.mertens@uza.be**

Antwerp University Hospital

Univ. Dept. Otorhinolaryngology, Head and Neck Surgery

Griet Mertens

Wilrijkstraat 10

2650 Edegem, Belgium

0032 38 21 32 45

### **Additional Information**

*Competing information:*

*Financial disclosure: The Antwerp University Hospital is currently receiving a research grant from MED-EL (Innsbruck, Austria). Non-Financial disclosure: None.*

**Table B online. Overview p_BTL_ values.**

*The length along the cochlea CDL_LW_ (Ɵ) as percentage length of the basal turn BTL_LW_. p_BTL_ values are retrieved from Schurzig et al. and are derived from 20 muCT datasets resulting in the equation
p_BTL_ (Ɵ) = 8.3 * 10^-8^ * Ɵ^3^ – 2.4 * 10^-4^ * Ɵ^2^ + 3.4 * 10^-1^ * Ɵ + 3.7.*

| **Angle [deg]** | **pBTL**  **[%]** | **Angle [deg]** | **pBTL**  **[%]** | **Angle [deg]** | **pBTL**  **[%]** | **Angle [deg]** | **pBTL**  **[%]** | **Angle [deg]** | **pBTL**  **[%]** | **Angle [deg]** | **pBTL**  **[%]** | **Angle [deg]** | **pBTL**  **[%]** | **Angle [deg]** | **pBTL**  **[%]** | **Angle [deg]** | **pBTL**  **[%]** | **Angle [deg]** | **pBTL**  **[%]** |
| --- | --- | --- | --- | --- | --- | --- | --- | --- | --- | --- | --- | --- | --- | --- | --- | --- | --- | --- | --- |
| 1 | 0.44 | 91 | 33.68 | 181 | 59.04 | 271 | 80.85 | 361 | 100.19 | 451 | 115.99 | 541 | 130.59 | 631 | 145.39 | 721 | 159.15 | 811 | 170.86 |
| 2 | 0.87 | 92 | 34.00 | 182 | 59.29 | 272 | 81.08 | 362 | 100.38 | 452 | 116.15 | 542 | 130.75 | 632 | 145.56 | 722 | 159.29 | 812 | 170.98 |
| 3 | 1.30 | 93 | 34.32 | 183 | 59.55 | 273 | 81.32 | 363 | 100.58 | 453 | 116.32 | 543 | 130.91 | 633 | 145.72 | 723 | 159.43 | 813 | 171.10 |
| 4 | 1.74 | 94 | 34.63 | 184 | 59.80 | 274 | 81.55 | 364 | 100.77 | 454 | 116.48 | 544 | 131.07 | 634 | 145.88 | 724 | 159.57 | 814 | 171.22 |
| 5 | 2.17 | 95 | 34.95 | 185 | 60.05 | 275 | 81.79 | 365 | 100.96 | 455 | 116.64 | 545 | 131.23 | 635 | 146.05 | 725 | 159.70 | 815 | 171.35 |
| 6 | 2.59 | 96 | 35.26 | 186 | 60.30 | 276 | 82.02 | 366 | 101.15 | 456 | 116.81 | 546 | 131.40 | 636 | 146.21 | 726 | 159.84 | 816 | 171.47 |
| 7 | 3.02 | 97 | 35.57 | 187 | 60.55 | 277 | 82.26 | 367 | 101.34 | 457 | 116.97 | 547 | 131.56 | 637 | 146.37 | 727 | 159.98 | 817 | 171.59 |
| 8 | 3.44 | 98 | 35.88 | 188 | 60.80 | 278 | 82.49 | 368 | 101.53 | 458 | 117.14 | 548 | 131.72 | 638 | 146.53 | 728 | 160.12 | 818 | 171.71 |
| 9 | 3.87 | 99 | 36.19 | 189 | 61.05 | 279 | 82.72 | 369 | 101.72 | 459 | 117.30 | 549 | 131.88 | 639 | 146.70 | 729 | 160.26 | 819 | 171.83 |
| 10 | 4.29 | 100 | 36.50 | 190 | 61.30 | 280 | 82.96 | 370 | 101.90 | 460 | 117.46 | 550 | 132.05 | 640 | 146.86 | 730 | 160.39 | 820 | 171.95 |
| 11 | 4.71 | 101 | 36.81 | 191 | 61.55 | 281 | 83.19 | 371 | 102.09 | 461 | 117.63 | 551 | 132.21 | 641 | 147.02 | 731 | 160.53 | 821 | 172.07 |
| 12 | 5.12 | 102 | 37.12 | 192 | 61.79 | 282 | 83.42 | 372 | 102.28 | 462 | 117.79 | 552 | 132.37 | 642 | 147.18 | 732 | 160.67 | 822 | 172.20 |
| 13 | 5.54 | 103 | 37.43 | 193 | 62.04 | 283 | 83.65 | 373 | 102.47 | 463 | 117.95 | 553 | 132.53 | 643 | 147.34 | 733 | 160.81 | 823 | 172.32 |
| 14 | 5.95 | 104 | 37.73 | 194 | 62.29 | 284 | 83.89 | 374 | 102.65 | 464 | 118.11 | 554 | 132.70 | 644 | 147.51 | 734 | 160.94 | 824 | 172.44 |
| 15 | 6.37 | 105 | 38.04 | 195 | 62.54 | 285 | 84.12 | 375 | 102.84 | 465 | 118.28 | 555 | 132.86 | 645 | 147.67 | 735 | 161.08 | 825 | 172.56 |
| 16 | 6.78 | 106 | 38.34 | 196 | 62.78 | 286 | 84.35 | 376 | 103.02 | 466 | 118.44 | 556 | 133.02 | 646 | 147.83 | 736 | 161.21 | 826 | 172.68 |
| 17 | 7.19 | 107 | 38.65 | 197 | 63.03 | 287 | 84.58 | 377 | 103.21 | 467 | 118.60 | 557 | 133.19 | 647 | 147.99 | 737 | 161.35 | 827 | 172.80 |
| 18 | 7.59 | 108 | 38.95 | 198 | 63.27 | 288 | 84.81 | 378 | 103.39 | 468 | 118.77 | 558 | 133.35 | 648 | 148.15 | 738 | 161.49 | 828 | 172.92 |
| 19 | 8.00 | 109 | 39.25 | 199 | 63.52 | 289 | 85.04 | 379 | 103.58 | 469 | 118.93 | 559 | 133.51 | 649 | 148.31 | 739 | 161.62 | 829 | 173.04 |
| 20 | 8.41 | 110 | 39.55 | 200 | 63.76 | 290 | 85.27 | 380 | 103.76 | 470 | 119.09 | 560 | 133.68 | 650 | 148.47 | 740 | 161.76 | 830 | 173.16 |
| 21 | 8.81 | 111 | 39.85 | 201 | 64.01 | 291 | 85.50 | 381 | 103.95 | 471 | 119.25 | 561 | 133.84 | 651 | 148.63 | 741 | 161.89 | 831 | 173.28 |
| 22 | 9.21 | 112 | 40.15 | 202 | 64.25 | 292 | 85.72 | 382 | 104.13 | 472 | 119.42 | 562 | 134.00 | 652 | 148.79 | 742 | 162.03 | 832 | 173.40 |
| 23 | 9.61 | 113 | 40.45 | 203 | 64.50 | 293 | 85.95 | 383 | 104.31 | 473 | 119.58 | 563 | 134.17 | 653 | 148.95 | 743 | 162.16 | 833 | 173.52 |
| 24 | 10.01 | 114 | 40.74 | 204 | 64.74 | 294 | 86.18 | 384 | 104.49 | 474 | 119.74 | 564 | 134.33 | 654 | 149.11 | 744 | 162.30 | 834 | 173.63 |
| 25 | 10.40 | 115 | 41.04 | 205 | 64.99 | 295 | 86.41 | 385 | 104.67 | 475 | 119.90 | 565 | 134.49 | 655 | 149.27 | 745 | 162.43 | 835 | 173.75 |
| 26 | 10.80 | 116 | 41.34 | 206 | 65.23 | 296 | 86.63 | 386 | 104.85 | 476 | 120.06 | 566 | 134.66 | 656 | 149.43 | 746 | 162.56 | 836 | 173.87 |
| 27 | 11.19 | 117 | 41.63 | 207 | 65.47 | 297 | 86.86 | 387 | 105.04 | 477 | 120.23 | 567 | 134.82 | 657 | 149.58 | 747 | 162.70 | 837 | 173.97 |
| 28 | 11.58 | 118 | 41.92 | 208 | 65.72 | 298 | 87.08 | 388 | 105.22 | 478 | 120.39 | 568 | 134.98 | 658 | 149.74 | 748 | 162.83 | 838 | 174.09 |
| 29 | 11.98 | 119 | 42.22 | 209 | 65.96 | 299 | 87.31 | 389 | 105.40 | 479 | 120.55 | 569 | 135.15 | 659 | 149.90 | 749 | 162.96 | 839 | 174.21 |
| 30 | 12.36 | 120 | 42.51 | 210 | 66.20 | 300 | 87.53 | 390 | 105.58 | 480 | 120.71 | 570 | 135.31 | 660 | 150.06 | 750 | 163.10 | 840 | 174.32 |
| 31 | 12.75 | 121 | 42.80 | 211 | 66.45 | 301 | 87.76 | 391 | 105.75 | 481 | 120.88 | 571 | 135.48 | 661 | 150.22 | 751 | 163.23 | 841 | 174.44 |
| 32 | 13.14 | 122 | 43.09 | 212 | 66.69 | 302 | 87.98 | 392 | 105.93 | 482 | 121.04 | 572 | 135.64 | 662 | 150.37 | 752 | 163.36 | 842 | 174.56 |
| 33 | 13.52 | 123 | 43.38 | 213 | 66.93 | 303 | 88.20 | 393 | 106.11 | 483 | 121.20 | 573 | 135.81 | 663 | 150.53 | 753 | 163.50 | 843 | 174.67 |
| 34 | 13.91 | 124 | 43.67 | 214 | 67.17 | 304 | 88.42 | 394 | 106.29 | 484 | 121.36 | 574 | 135.97 | 664 | 150.69 | 754 | 163.63 | 844 | 174.79 |
| 35 | 14.29 | 125 | 43.96 | 215 | 67.42 | 305 | 88.65 | 395 | 106.47 | 485 | 121.52 | 575 | 136.13 | 665 | 150.84 | 755 | 163.76 | 845 | 174.44 |
| 36 | 14.67 | 126 | 44.24 | 216 | 67.66 | 306 | 88.87 | 396 | 106.64 | 486 | 121.69 | 576 | 136.30 | 666 | 151.00 | 756 | 163.89 | 846 | 174.56 |
| 37 | 15.05 | 127 | 44.53 | 217 | 67.90 | 307 | 89.09 | 397 | 106.82 | 487 | 121.85 | 577 | 136.46 | 667 | 151.16 | 757 | 164.02 | 847 | 174.67 |
| 38 | 15.42 | 128 | 44.82 | 218 | 68.14 | 308 | 89.31 | 398 | 107.00 | 488 | 122.01 | 578 | 136.63 | 668 | 151.31 | 758 | 164.15 | 848 | 174.79 |
| 39 | 15.80 | 129 | 45.10 | 219 | 68.39 | 309 | 89.53 | 399 | 107.17 | 489 | 122.17 | 579 | 136.79 | 669 | 151.47 | 759 | 164.29 | 849 | 174.90 |
| 40 | 16.17 | 130 | 45.38 | 220 | 68.63 | 310 | 89.75 | 400 | 107.35 | 490 | 122.33 | 580 | 136.96 | 670 | 151.62 | 760 | 164.42 | 850 | 175.02 |
| 41 | 16.55 | 131 | 45.67 | 221 | 68.87 | 311 | 89.96 | 401 | 107.53 | 491 | 122.50 | 581 | 137.12 | 671 | 151.78 | 761 | 164.55 | 851 | 175.13 |
| 42 | 16.92 | 132 | 45.95 | 222 | 69.11 | 312 | 90.18 | 402 | 107.70 | 492 | 122.66 | 582 | 137.29 | 672 | 151.93 | 762 | 164.68 | 852 | 175.25 |
| 43 | 17.29 | 133 | 46.23 | 223 | 69.35 | 313 | 90.40 | 403 | 107.88 | 493 | 122.82 | 583 | 137.45 | 673 | 152.09 | 763 | 164.81 | 853 | 175.25 |
| 44 | 17.66 | 134 | 46.51 | 224 | 69.59 | 314 | 90.62 | 404 | 108.05 | 494 | 122.98 | 584 | 137.62 | 674 | 152.24 | 764 | 164.94 | 854 | 175.36 |
| 45 | 18.03 | 135 | 46.79 | 225 | 69.84 | 315 | 90.83 | 405 | 108.23 | 495 | 123.14 | 585 | 137.79 | 675 | 152.40 | 765 | 165.07 | 855 | 175.48 |
| 46 | 18.39 | 136 | 47.07 | 226 | 70.08 | 316 | 91.05 | 406 | 108.40 | 496 | 123.31 | 586 | 137.95 | 676 | 152.55 | 766 | 165.20 | 856 | 175.59 |
| 47 | 18.76 | 137 | 47.35 | 227 | 70.32 | 317 | 91.26 | 407 | 108.58 | 497 | 123.47 | 587 | 138.12 | 677 | 152.70 | 767 | 165.33 | 857 | 175.70 |
| 48 | 19.12 | 138 | 47.63 | 228 | 70.56 | 318 | 91.48 | 408 | 108.75 | 498 | 123.63 | 588 | 138.28 | 678 | 152.86 | 768 | 165.46 | 858 | 175.81 |
| 49 | 19.48 | 139 | 47.91 | 229 | 70.80 | 319 | 91.69 | 409 | 108.92 | 499 | 123.79 | 589 | 138.45 | 679 | 153.01 | 769 | 165.59 | 859 | 175.93 |
| 50 | 19.84 | 140 | 48.19 | 230 | 71.04 | 320 | 91.90 | 410 | 109.10 | 500 | 123.95 | 590 | 138.61 | 680 | 153.16 | 770 | 165.72 | 860 | 176.04 |
| 51 | 20.20 | 141 | 48.46 | 231 | 71.28 | 321 | 92.11 | 411 | 109.27 | 501 | 124.12 | 591 | 138.78 | 681 | 153.31 | 771 | 165.84 | 861 | 176.15 |
| 52 | 20.56 | 142 | 48.74 | 232 | 71.52 | 322 | 92.33 | 412 | 109.44 | 502 | 124.28 | 592 | 138.94 | 682 | 153.46 | 772 | 165.97 | 862 | 176.26 |
| 53 | 20.92 | 143 | 49.02 | 233 | 71.77 | 323 | 92.54 | 413 | 109.61 | 503 | 124.44 | 593 | 139.11 | 683 | 153.62 | 773 | 166.10 | 863 | 176.37 |
| 54 | 21.28 | 144 | 49.29 | 234 | 72.01 | 324 | 92.75 | 414 | 109.79 | 504 | 124.60 | 594 | 139.28 | 684 | 153.77 | 774 | 166.23 | 864 | 176.48 |
| 55 | 21.63 | 145 | 49.56 | 235 | 72.25 | 325 | 92.96 | 415 | 109.96 | 505 | 124.76 | 595 | 139.44 | 685 | 153.92 | 775 | 166.36 | 865 | 176.59 |
| 56 | 21.98 | 146 | 49.84 | 236 | 72.49 | 326 | 93.17 | 416 | 110.13 | 506 | 124.93 | 596 | 139.61 | 686 | 154.07 | 776 | 166.49 | 866 | 176.70 |
| 57 | 22.34 | 147 | 50.11 | 237 | 72.73 | 327 | 93.38 | 417 | 110.30 | 507 | 125.09 | 597 | 139.77 | 687 | 154.22 | 777 | 166.61 | 867 | 176.81 |
| 58 | 22.69 | 148 | 50.38 | 238 | 72.97 | 328 | 93.59 | 418 | 110.47 | 508 | 125.25 | 598 | 139.94 | 688 | 154.37 | 778 | 166.74 | 868 | 176.92 |
| 59 | 23.04 | 149 | 50.66 | 239 | 73.21 | 329 | 93.79 | 419 | 110.64 | 509 | 125.41 | 599 | 140.10 | 689 | 154.52 | 779 | 166.87 | 869 | 177.03 |
| 60 | 23.39 | 150 | 50.93 | 240 | 73.45 | 330 | 94.00 | 420 | 110.81 | 510 | 125.57 | 600 | 140.27 | 690 | 154.67 | 780 | 167.00 | 870 | 177.14 |
| 61 | 23.73 | 151 | 51.20 | 241 | 73.69 | 331 | 94.21 | 421 | 110.98 | 511 | 125.73 | 601 | 140.44 | 691 | 154.82 | 781 | 167.12 | 871 | 177.25 |
| 62 | 24.08 | 152 | 51.47 | 242 | 73.93 | 332 | 94.41 | 422 | 111.15 | 512 | 125.90 | 602 | 140.60 | 692 | 154.97 | 782 | 167.25 | 872 | 177.36 |
| 63 | 24.42 | 153 | 51.74 | 243 | 74.17 | 333 | 94.62 | 423 | 111.32 | 513 | 126.06 | 603 | 140.77 | 693 | 155.12 | 783 | 167.38 | 873 | 177.47 |
| 64 | 24.77 | 154 | 52.01 | 244 | 74.41 | 334 | 94.83 | 424 | 111.49 | 514 | 126.22 | 604 | 140.93 | 694 | 155.26 | 784 | 167.50 | 874 | 177.58 |
| 65 | 25.11 | 155 | 52.27 | 245 | 74.65 | 335 | 95.03 | 425 | 111.66 | 515 | 126.38 | 605 | 141.10 | 695 | 155.41 | 785 | 167.63 | 875 | 177.69 |
| 66 | 25.45 | 156 | 52.54 | 246 | 74.89 | 336 | 95.24 | 426 | 111.83 | 516 | 126.54 | 606 | 141.27 | 696 | 155.56 | 786 | 167.75 | 876 | 177.79 |
| 67 | 25.79 | 157 | 52.81 | 247 | 75.13 | 337 | 95.44 | 427 | 112.00 | 517 | 126.71 | 607 | 141.43 | 697 | 155.71 | 787 | 167.88 | 877 | 177.90 |
| 68 | 26.13 | 158 | 53.08 | 248 | 75.37 | 338 | 95.64 | 428 | 112.17 | 518 | 126.87 | 608 | 141.60 | 698 | 155.85 | 788 | 168.01 | 878 | 178.01 |
| 69 | 26.47 | 159 | 53.34 | 249 | 75.61 | 339 | 95.85 | 429 | 112.34 | 519 | 127.03 | 609 | 141.76 | 699 | 156.00 | 789 | 168.13 | 879 | 178.12 |
| 70 | 26.81 | 160 | 53.61 | 250 | 75.85 | 340 | 96.05 | 430 | 112.51 | 520 | 127.19 | 610 | 141.93 | 700 | 156.15 | 790 | 168.26 | 880 | 178.22 |
| 71 | 27.15 | 161 | 53.87 | 251 | 76.09 | 341 | 96.25 | 431 | 112.67 | 521 | 127.35 | 611 | 142.10 | 701 | 156.29 | 791 | 168.38 | 881 | 178.33 |
| 72 | 27.48 | 162 | 54.14 | 252 | 76.33 | 342 | 96.45 | 432 | 112.84 | 522 | 127.51 | 612 | 142.26 | 702 | 156.44 | 792 | 168.51 | 882 | 178.43 |
| 73 | 27.82 | 163 | 54.40 | 253 | 76.57 | 343 | 96.65 | 433 | 113.01 | 523 | 127.68 | 613 | 142.43 | 703 | 156.58 | 793 | 168.63 | 883 | 178.54 |
| 74 | 28.15 | 164 | 54.66 | 254 | 76.81 | 344 | 96.85 | 434 | 113.18 | 524 | 127.84 | 614 | 142.59 | 704 | 156.73 | 794 | 168.76 | 884 | 178.64 |
| 75 | 28.49 | 165 | 54.92 | 255 | 77.05 | 345 | 97.05 | 435 | 113.34 | 525 | 128.00 | 615 | 142.76 | 705 | 156.87 | 795 | 168.88 | 885 | 178.75 |
| 76 | 28.82 | 166 | 55.19 | 256 | 77.29 | 346 | 97.25 | 436 | 113.51 | 526 | 128.16 | 616 | 142.92 | 706 | 157.02 | 796 | 169.01 | 886 | 178.85 |
| 77 | 29.15 | 167 | 55.45 | 257 | 77.53 | 347 | 97.45 | 437 | 113.68 | 527 | 128.32 | 617 | 143.09 | 707 | 157.16 | 797 | 169.13 | 887 | 178.96 |
| 78 | 29.48 | 168 | 55.71 | 258 | 77.76 | 348 | 97.65 | 438 | 113.84 | 528 | 128.48 | 618 | 143.25 | 708 | 157.31 | 798 | 169.26 | 888 | 179.06 |
| 79 | 29.81 | 169 | 55.97 | 259 | 78.00 | 349 | 97.85 | 439 | 114.01 | 529 | 128.65 | 619 | 143.42 | 709 | 157.45 | 799 | 169.38 | 889 | 179.17 |
| 80 | 30.14 | 170 | 56.23 | 260 | 78.24 | 350 | 98.05 | 440 | 114.18 | 530 | 128.81 | 620 | 143.58 | 710 | 157.59 | 800 | 169.50 | 890 | 179.27 |
| 81 | 30.46 | 171 | 56.49 | 261 | 78.48 | 351 | 98.25 | 441 | 114.34 | 531 | 128.97 | 621 | 143.75 | 711 | 157.73 | 801 | 169.63 | 891 | 179.37 |
| 82 | 30.79 | 172 | 56.74 | 262 | 78.72 | 352 | 98.44 | 442 | 114.51 | 532 | 129.13 | 622 | 143.91 | 712 | 157.88 | 802 | 169.75 | 892 | 179.47 |
| 83 | 31.12 | 173 | 57.00 | 263 | 78.95 | 353 | 98.64 | 443 | 114.67 | 533 | 129.29 | 623 | 144.08 | 713 | 158.02 | 803 | 169.88 | 893 | 179.58 |
| 84 | 31.44 | 174 | 57.26 | 264 | 79.19 | 354 | 98.83 | 444 | 114.84 | 534 | 129.45 | 624 | 144.24 | 714 | 158.16 | 804 | 170.00 | 894 | 179.09 |
| 85 | 31.76 | 175 | 57.51 | 265 | 79.43 | 355 | 99.03 | 445 | 115.00 | 535 | 129.62 | 625 | 144.41 | 715 | 158.30 | 805 | 170.12 | 895 | 179.19 |
| 86 | 32.09 | 176 | 57.77 | 266 | 79.67 | 356 | 99.22 | 446 | 115.17 | 536 | 129.78 | 626 | 144.57 | 716 | 158.44 | 806 | 170.24 | 896 | 179.29 |
| 87 | 32.41 | 177 | 58.03 | 267 | 79.90 | 357 | 99.42 | 447 | 115.33 | 537 | 129.94 | 627 | 144.74 | 717 | 158.59 | 807 | 170.37 | 897 | 179.39 |
| 88 | 32.73 | 178 | 58.28 | 268 | 80.14 | 358 | 99.61 | 448 | 115.50 | 538 | 130.10 | 628 | 144.90 | 718 | 158.73 | 808 | 170.49 | 898 | 179.50 |
| 89 | 33.05 | 179 | 58.53 | 269 | 80.38 | 359 | 99.81 | 449 | 115.66 | 539 | 130.26 | 629 | 145.07 | 719 | 158.87 | 809 | 170.61 | 899 | 179.60 |
| 90 | 33.37 | 180 | 58.79 | 270 | 80.61 | 360 | 100.00 | 450 | 115.83 | 540 | 130.42 | 630 | 145.23 | 720 | 159.01 | 810 | 170.74 | 900 | 179.70 |
